# Enhancing cryo-EM maps with 3D deep generative networks for assisting protein structure modeling

**DOI:** 10.1093/bioinformatics/btad494

**Published:** 2023-08-07

**Authors:** Sai Raghavendra Maddhuri Venkata Subramaniya, Genki Terashi, Daisuke Kihara

**Affiliations:** Department of Computer Science, Purdue University, West Lafayette, IN 47907, United States; Department of Biological Sciences, Purdue University, West Lafayette, IN 47907, United States; Department of Computer Science, Purdue University, West Lafayette, IN 47907, United States; Department of Biological Sciences, Purdue University, West Lafayette, IN 47907, United States

## Abstract

**Motivation:**

The tertiary structures of an increasing number of biological macromolecules have been determined using cryo-electron microscopy (cryo-EM). However, there are still many cases where the resolution is not high enough to model the molecular structures with standard computational tools. If the resolution obtained is near the empirical borderline (3–4.5 Å), improvement in the map quality facilitates structure modeling.

**Results:**

We report EM-GAN, a novel approach that modifies an input cryo-EM map to assist protein structure modeling. The method uses a 3D generative adversarial network (GAN) that has been trained on high- and low-resolution density maps to learn the density patterns, and modifies the input map to enhance its suitability for modeling. The method was tested extensively on a dataset of 65 EM maps in the resolution range of 3–6 Å and showed substantial improvements in structure modeling using popular protein structure modeling tools.

**Availability and implementation:**

https://github.com/kiharalab/EM-GAN, Google Colab: https://tinyurl.com/3ccxpttx.

## 1 Introduction

Technological advances in cryo-EM have led to its rapid adoption in determining structures of biological macromolecules, including structures that were challenging to determine by other experimental methods. With improvements in software and hardware, an increasing number of structures can now be determined at atomic or near-atomic resolutions. However, many maps are still determined at lower resolutions that pose challenges for structure modeling (Wang *et al.* 2015, Chen *et al.* 2016, Terashi and Kihara 2018a,b, Terwilliger *et al.* 2018). As a result, structure modeling of macromolecules becomes increasingly difficult, especially for maps in the critical resolution range of 3–4.5 Å.

Several methods have been developed that post-process cryo-EM maps to aid in interpretation. Map sharpening based on B-factor correction (Rosenthal and Henderson 2003) is routinely applied in the structure modeling pipeline. LocScale (Jakobi *et al.* 2017) makes use of solved atomic models to upscale the map densities. LocalDeblur (Ramírez-Aportela *et al.* 2020) performs local map sharpening based on a model that correlates pairs of experimental and sharpened maps. Terwilliger *et al.* developed a maximum-likelihood approach for density modification from half maps (Terwilliger *et al.* 2020). CryoDRGN (Zhong *et al.* 2021), CryoGAN (Gupta *et al.* 2021), and Topaz-Denoise (Bepler *et al.* 2020) applied generative models to 2D particle images of cryo-EM maps. OPUS-SSRI incorporates sparseness and smoothness into optimization process during map refinement (Luo *et al.* 2021).

Deep learning (DL) has been applied in processing cryo-EM maps. We previously developed Emap2sec, which applied DL to cryo-EM maps for secondary structure and nucleic acid detection (Subramaniya *et al.* 2019). DeepTracer uses DL to predict local protein structures from map density and incorporate these predictions into protein structure modeling (Pfab *et al.* 2021). CryoGAN is an exploratory work, which uses a generative adversarial network (GAN) (Goodfellow *et al.* 2014) to construct a 3D EM map from 2D particle images. DeepEMhancer used DL to sharpen density maps by learning from maps sharpened by existing software (Sanchez-Garcia *et al.* 2021). The current work, EM-GAN, is for modifying an input 3D EM map to assist protein structure modeling by using maps simulated from protein atomic structures as targets. This makes EM-GAN distinct from other existing works in terms of the target maps used for training and its intended purpose.

Here, we developed a novel method, EM-GAN, which applies GAN, a type of DL architecture, directly to 3D density maps to improve the structure modeling capability of experimental EM maps in the range of 3–6 Å resolution. GAN has been successfully applied to super-resolution tasks for photographs and other image data. Super-resolution imaging involves generating high-resolution images from low-resolution inputs (Yang *et al.* 2014). With advancements in DL, the recent introduction of GAN has achieved state-of-the-art results in image resolution improvement (Ledig *et al.* 2017). GAN has been successfully applied to various related tasks, such as improving medical images (Ker *et al.* 2018, Yang *et al.* 2018) and restoring images (Lehtinen *et al.* 2018). Super-resolution imaging applications of GANs have also been extended to other microscopy image data, such as from fluorescence microscopy (Wang *et al.* 2019), single-molecule localization microscopy (Ouyang *et al.* 2018), MRI (Sánchez *et al*. 2018), and 2D computed tomography (CT) scan images (Yang *et al.* 2018). Unlike these existing GAN applications to medical and biological images, EM-GAN is applied specifically to 3D low-resolution images.

In EM-GAN, as the end goal is to improve structure modeling from maps, we used simulated maps derived from protein atomic structures for training. We trained the networks to modify low-resolution input maps into outputs that are indistinguishable from actual high-resolution simulated maps. Our results demonstrate that the maps modified by EM-GAN indeed led to better protein structure models when structures were modeled by Phenix (Terwilliger *et al.* 2018) and MAINMAST (Terashi and Kihara 2018). Structure models generated by MAINMAST and Phenix have shown an average amino acid coverage improvement of 2.57% and 6.87%, respectively, when using modified maps generated by EM-GAN. We also compared EM-GAN with other state-of-the-art methods, namely OPUS-SSRI and DeepEMhancer, and showed that EM-GAN performed better in terms of both the resolution of modified maps and the accuracy of protein structure modeling using the modified maps.

## 2 Materials and methods

### 2.1 Dataset generation

EM-GAN required a dataset of EM density maps, experimental and high-resolution simulated maps, for training. We extracted cubes from experimental EM maps and paired them with corresponding simulated EM maps derived from the associated atomic-detailed structures available in the PDB as high-resolution data.

The dataset of experimental maps was generated using a procedure similar to that used in developing our past work, Emap2sec (Subramaniya *et al.* 2019). Specifically, for the experimental map dataset, we obtained EM maps from EMDB as follows: 788 EM maps with resolutions between 3 and 6 Å and associated structures in PDB were initially selected. Associated atomic coordinate structures were necessary so that corresponding simulated EM maps could be generated. To ensure that the maps had a good correspondence with their corresponding PDB structures, we filtered the initial set of 788 maps to only include experimental maps with a cross-correlation coefficient of 0.65 or higher with the simulated map generated from the corresponding PDB structure. This resulted in a final dataset of 377 maps. To eliminate redundant examples, we removed maps that had proteins with over 25% sequence identity to any protein in another map in the dataset. This procedure finally yielded a pool of 122 experimental EM maps. The experimental maps were converted to a uniform grid spacing of 1.0 Å through trilinear interpolation. High-resolution simulated EM maps were then generated from the PDB structures of the above 122 experimental maps using the pdb2vol program from the SITUS package (Wriggers 2012). For experimental maps with resolutions ranging from 3.5 to 6.0 Å, the corresponding high-resolution simulated maps were generated at a resolution of 3.0 Å. Conversely, for experimental maps with resolutions between 3.0 and 3.5 Å, the corresponding high-resolution simulated maps were generated at a resolution of 1.8 Å.

In both simulated and experimental maps, electron density values were normalized from 0.0 to 1.0 by subtracting the minimum density value in the map and then dividing by the difference between the maximum and the minimum density values. If there were negative density values, they were set to 0.0 before the normalization. Each of the EM maps was then converted to smaller chunks by a sliding cube of size 25^3^ Å^3^ translated through the maps with a stride size of 4 Å. This procedure generated a total of 292 749 valid pairs of low- and high-resolution cubes. A cube pair was considered valid if neither cube contained all-zero density values. Out of the 122 EM map pairs generated, 86 pairs, amounting to 201, 552 cube pairs were used as the training and validation set, and 36 pairs, amounting to 91 197 cube pairs, were used for testing. For testing, we additionally included 29 maps with the same resolution range, which were previously used in the MAINMAST dataset yielding a testing set of 65 total maps with 45 311 cube pairs. Among them, 26 maps have a resolution better than or equal to 3.5 Å and 39 maps had a resolution worse than 3.5 Å.

### 2.2 Architecture of EM-GAN

EM-GAN takes an experimental 3D cryo-EM map as input and produces a modified map as output. The network architecture used was similar to SRGAN (Ledig *et al.* 2017). In general, a GAN is trained on pairs of a low-resolution image and a corresponding high-resolution image, so that the network learns how to modify a low-resolution image toward the paired high-resolution image. Following this idea, we trained EM-GAN on a dataset of pairs of an experimental EM map and a corresponding simulated EM map computed from the associated atomic-detail protein structure, so that the network will be able to modify an input experimental map toward the high-resolution simulated map. We set up the dataset in this way since the end goal is to produce higher quality protein structure models from maps.

**Figure 1.**
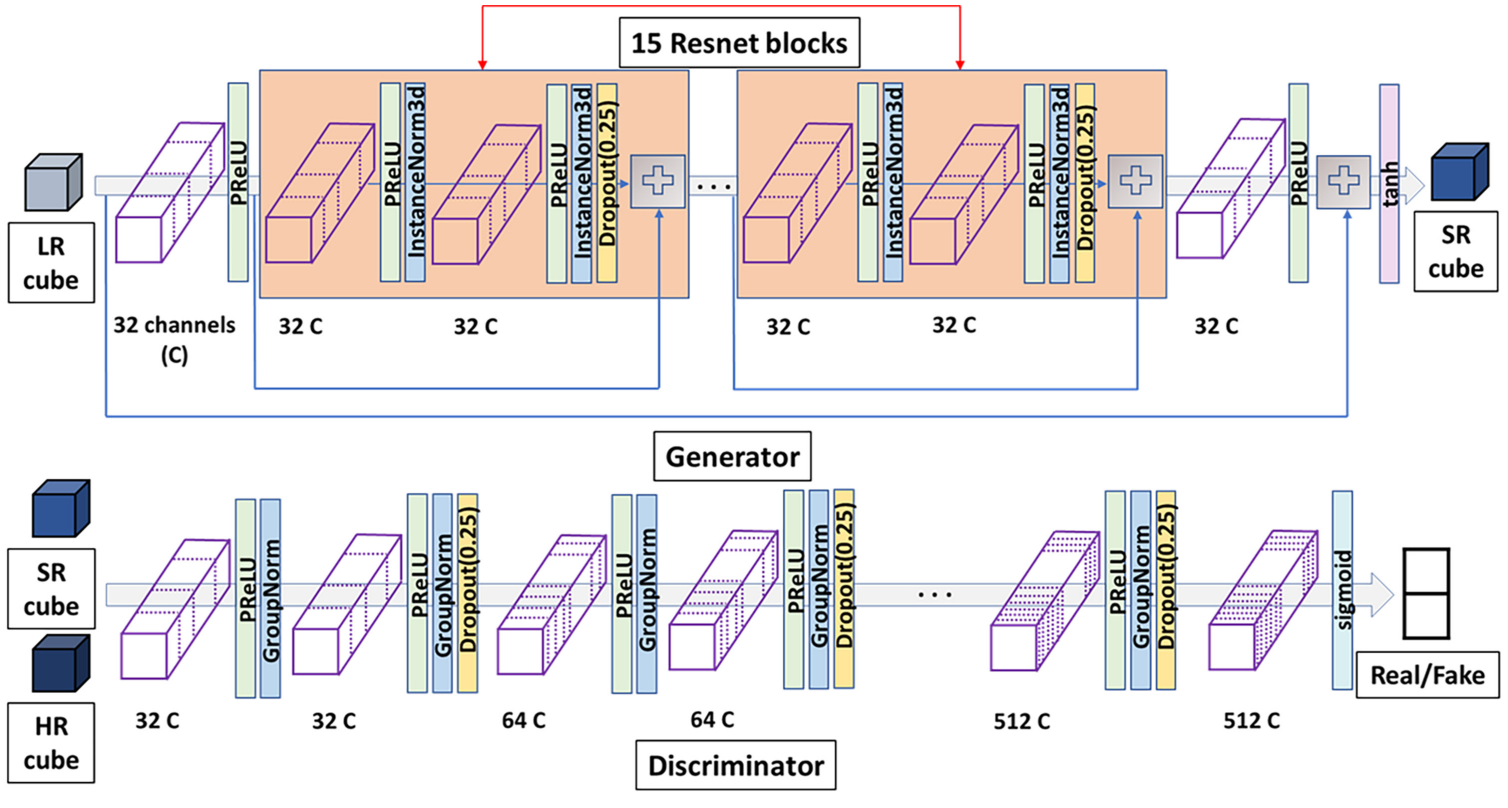
The 3D GAN architecture of EM-GAN. The detailed architecture of the generator (top panel) and discriminator (bottom panel) networks are shown. Exp., original experimental map; Mod., GAN-modified map; Sim, simulated map from atomic structure; PReLU, the parametric rectified linear unit activation function; InstanceNorm3D, 3D instance normalization layer; GroupNorm, group normalization layer; plus signs, tensor addition operations. The arrows that each connect the input of a ResNet block and a plus sign each represent a skip connection.

Figure 1 illustrates the architecture of EM-GAN. The inputs are a pair of cubes of a volume size of 25^3^ Å^3^ each, which are extracted by scanning EM maps. The generator module of the GAN takes a cube from an experimental map (Exp. Cube in Fig. 1) and outputs a GAN-modified density cube of the same size (Mod. Cube). Individual modified density cubes output from the network are merged to generate a complete EM map. The generator network is a fully convolutional network consisting of a 3D convolution layer with 32 channels and a kernel size of 3 followed by 15 residual network (ResNet) block layers, and a 3D convolution layer ending with a tanh activation layer. Each ResNet block contains two 3D convolutional layers with 32 channels and skip connections, dropout with a probability of 0.25, PReLU activations, and Instance Normalization, which was shown to work better than other normalization methods for generative tasks. The final 3D convolutional layer also uses a kernel size of 3 with 32 output channels. The last tanh layer outputs a probability distribution for each voxel in the cube, which is interpreted as the density distribution in the cube. The output of the tanh function is normalized to produce values in the range of 0 to 1. A stride of 1 was used in all the filters in all the layers.

The discriminator network, shown in the bottom panel in Fig. 1, is a fully convolutional binary classifier. It takes a generated cube output from the generator and the corresponding simulated density cube and classifies the two cubes into classes, real (simulated) or GAN-generated. The discriminator network consists of 10 3D CNNs followed by a softmax layer. The first convolutional layer in the discriminator contains 32 channels, and the count is multiplied by a factor of 2 for every two subsequent layers. We use PReLU, dropout with a probability of 0.25 and group normalization with a group size of 16 in all the convolutional layers.

### 2.3 Training procedure of EM-GAN

The training cube pairs described above were used to train the GAN network of EM-GAN. The input to the generator network of the GAN is the cube from the experimental map, referred to as the exp. cube. The output cube of the generator network and the corresponding cube from a simulated map cube were then fed to the discriminator network.

In GANs, the generator and discriminator are trained together with a minimax game-style objective function: where G and D are generator and discriminator networks of GAN, $P_{d}$ is the simulated map cube distribution, and ${\;P}_{g}$ is the generated cube distribution. The generator receives an exp. cube as input and generates a modified cube. The discriminator then classifies the modified cube and the corresponding high-resolution simulated cube. The minimax objective ensures that the generator generates superior quality modified cubes so that the generator can fool the discriminator into classifying them as simulated high-resolution cubes. The objective function for the generator of EM-GAN is formulated in Equation 2 as a linear combination of content loss and adversarial loss:


(1) min G maxD⁡(Ex ∼ Pdlog⁡Dx + Ez ∼ Pg[log⁡ (1-D(z))]) 

(2) $${\mathrm{Loss}}_{\mathrm{Generator}\;}={\mathrm{Loss}}_{\mathrm{content}\;}+\;10^{-3}\;{\mathrm{Loss}}_{\mathrm{adversarial}\;},\;$$

(3) $$\mathrm{where},\;{\mathrm{Loss}}_{\mathrm{content}\;}=\;\sum_{i=1}^{25}\sum_{j=1}^{25}{\sum_{k=1}^{25}\left({\mathrm{Sim}}_{i,j,k}-{\left(G\left(\;\text{Exp}\;\right)\right)}_{i,j,k}\right)}^{2}\;$$

(4) $$\mathrm{and}\;\;{\mathrm{Loss}}_{\mathrm{adversarial}\;}=\;-D(G\left(\;\text{Exp}\;\right))\;$$

*Sim* corresponds to the high-resolution cube, *Exp* corresponds to the experimental map cube, which makes the modified cube, *G(Exp)*, and *D(G(Exp))* is the discriminator probability output for the modified density cube ranging from 0 to 1. Negative of *D(G(Exp))* was optimized to fool the discriminator into thinking that modified cube is as good as the simulated cube. To summarize, the content loss is defined by the mean squared error between the modified cube and the simulated cube. The adversarial loss is given as the negative softmax probabilities of the discriminator predictions. The loss function of the discriminator is for the network to distinguish the two types of maps:


(5) $${\mathrm{Loss}}_{\mathrm{Discriminator}\;}=\left(1-D\left(x\right)\right)+D(G\left(\;\text{Exp}\;\right))$$

We experimented with four different learning rate values of (0.01, 0.001, 0.005, 0.0001) using a held-out validation set of 20 maps and used 66 maps for training. We chose a learning rate of 0.001 for both the generator and the discriminator based on the best validation performance. We used a batch size of 16 and trained the GAN network for 100 epochs using the Adam optimizer for updating the weights of both the generator and the discriminator. The computational time taken for inference is provided in Supplementary Table S3.

### 2.4 Protein structure modeling from maps

The structure modeling was performed separately on the experimental and modified maps using MAINMAST and Phenix. For MAINMAST, we first ran the protocol up to the main-chain generation step where MAINMAST generates 3600 models with different parameter combinations. These models were then sent to full-atom structure building and structure refinement by PULCHRA (Rotkiewicz and Skolnick 2008) and MDFF (McGreevy *et al.* 2016) and finally ranked by the scoring function of MDFF.

For Phenix, we used the map_to_model program, which takes an EM map, the target protein sequence, and a map resolution information to build a full atomic model. For the map resolution information to run map_to_model, we used the value reported in EMDB.

## 3 Results

We measured multiple aspects of the impact of the maps generated by EM-GAN on a dataset of 65 EM maps.

### 3.1 Cross-correlation change of maps

**Figure 2.**
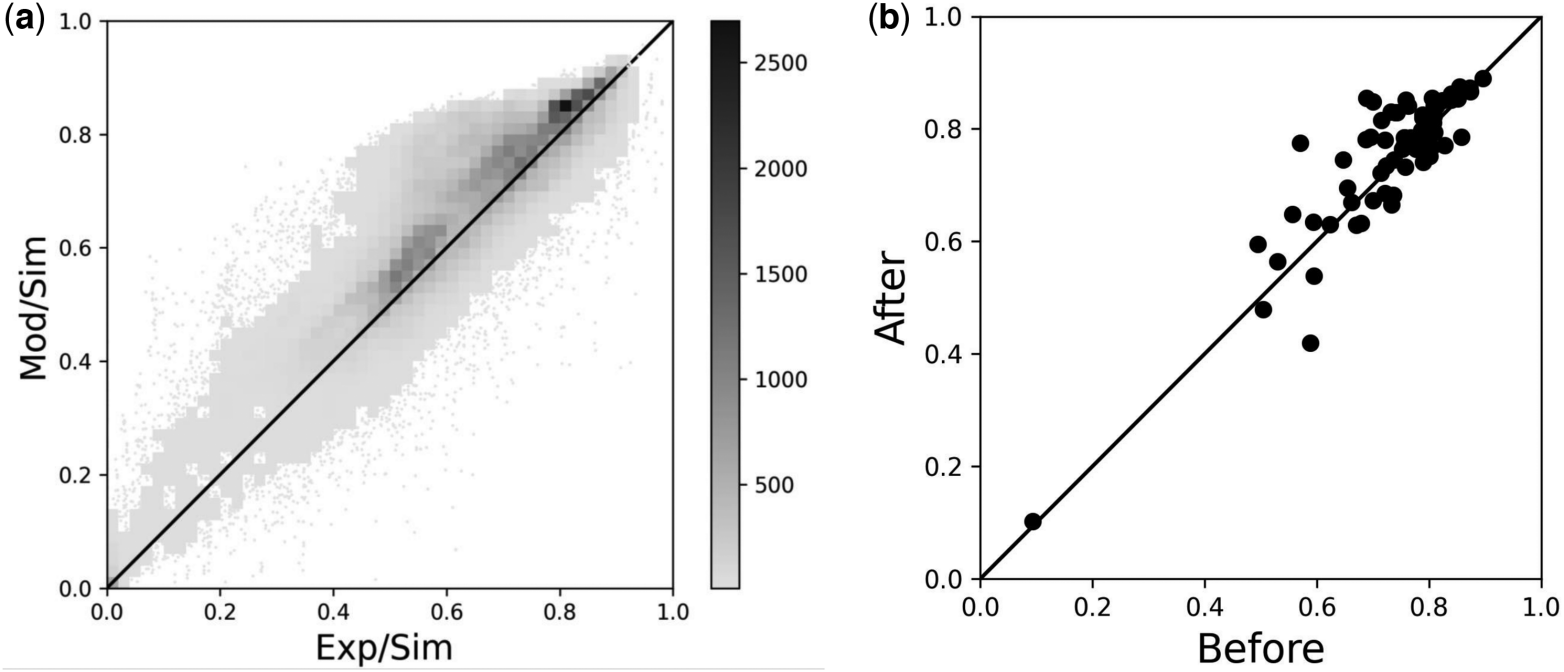
Local and residue-level map improvement by EM-GAN. (a) Local improvement. Cross-correlation of experimental & simulated (Exp/Sim) versus GAN-modified & simulated (Mod/Sim) of local 136 508 cubes (65 test maps) is shown. Gray scale bar indicates the number of points in each of the 50×50 bins in the plot. (b) Residue-wise correlation improvement through real-space correlation coefficient (RSCC). The estimated RSCC values before and after EM-GAN.

We first evaluated the changes of density maps produced by EM-GAN at the local cube level. Our data generation procedure, as described earlier, generated 136 508 cubes of size 25^3^ Å from the 65 test maps. These cubes from experimental maps are fed to EM-GAN, which generated the same number of corresponding modified cubes. For reference, we generated cubes from the simulated maps, which were computed from atomic structures in PDB. We then calculated cross-correlation between experimental/simulated cubes and GAN-modified/simulated cubes using the Chimera’s “measure correlation” tool (Pettersen *et al.* 2004) (Fig. 2a).

Figure 2a shows that, overall, EM-GAN increased the cross-correlation of cubes for most of the cases. An increase or same cross-correlation was observed for 77.0% (105129/136508) of the test cubes with an average improvement from 0.637 to 0.678. On average, 89.4% of cubes in a given map saw improved correlation through EM-GAN.

Next, we validated the density change of maps on a residue level. In Fig. 2b, we generated full size GAN-modified maps by combining modified cubes. Average density values were assigned for overlaps between cubes. To quantify the change made by EM-GAN, we computed the real-space correlation coefficient (RSCC) (Bränd’en and Alwyn Jones 1990, Tickle 2012), which evaluates the fit between a map and a corresponding model. RSCC provides a residue-wise correlation score and is thus suitable for assessing how well the backbone residues of the protein structure fit into a map. RSCC values were calculated between the experimental map versus the native structure and the corresponding modified maps generated by EM-GAN versus the native structure using the phenix.map_model_cc tool (Afonine *et al.* 2018) in the Phenix package (Liebschner *et al.* 2019). It is apparent from Fig. 2b that the RSCC values of GAN-modified map versus native structure were higher than or the same as the experimental map versus native structure for the majority of the cases (44 out of 65 maps). The average RSCC values increased from 0.728 to 0.748 (*P*-value of 0.0015 by *t*-test).

**Figure 3.**
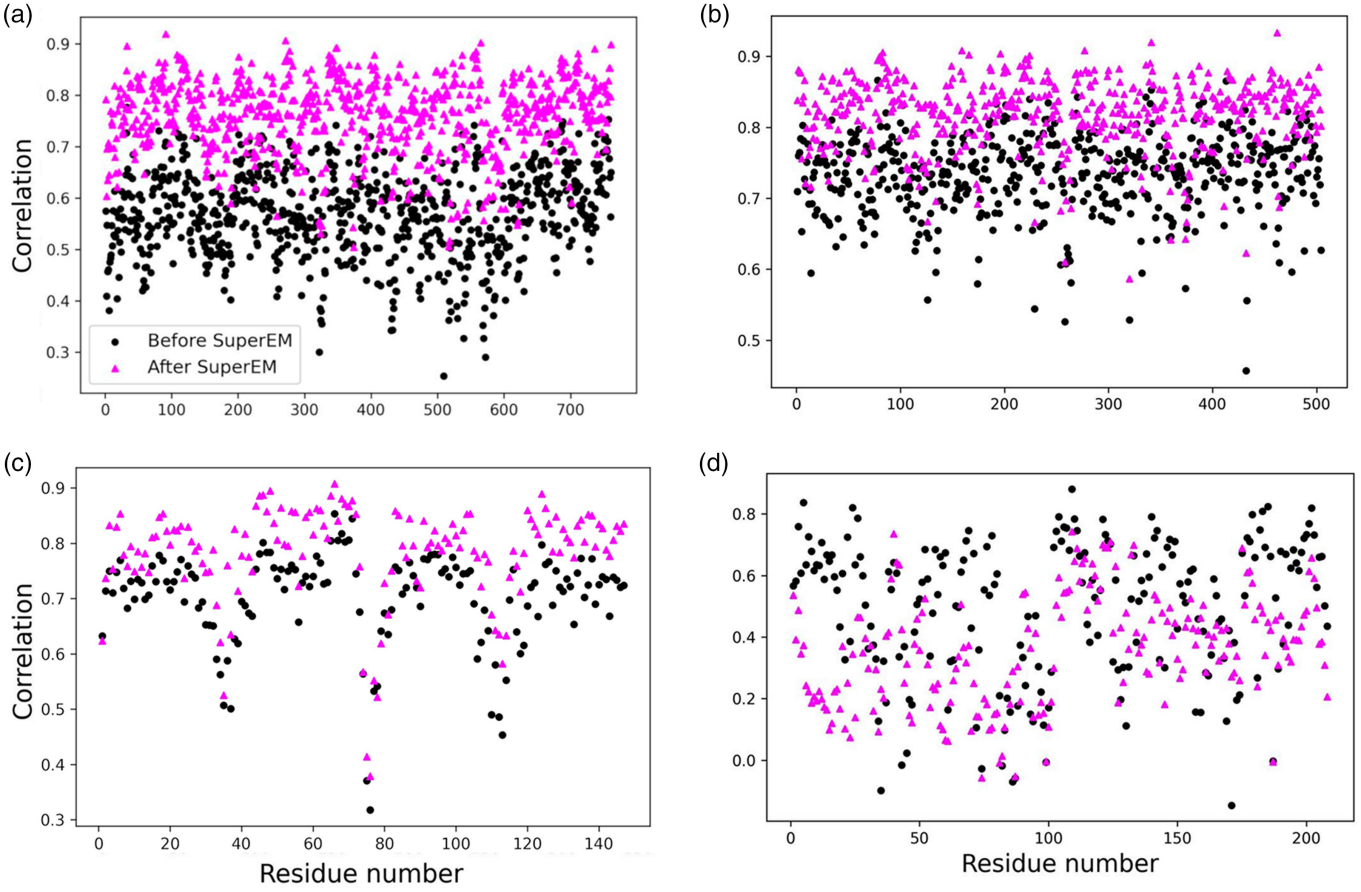
Examples of RSCC values of residues before and after EM-GAN application. Triangles show RSCC for the modified map by EM-GAN. Dots are RSCC of the experimental map. (a) Chain A of bacteriophage phi6 capsid (EMD-2364; PDB ID: 4btg). Average RSCC before EM-GAN: 0.570, after EM-GAN: 0.775. (b) Chain B of grapevine fanleaf virus complex with nanobody (EMD-3246, PDB ID: 5foj). Average RSCC before: 0.687, after: 0.781. (c) Chain D of membrane-embedded motor of a eukaryotic V-ATPase (EMD-8409, PDB ID: 5tj5). Average RSCC before: 0.712, after: 0.809. (d) chain L of trimeric HIV-1 envelope glycoprotein (EMD-2484, PDB ID: 4cc8L). Average RSCC before: 0.539, after: 0.330.

In Fig. 3, we show four examples of changes of RSCC. The first example (Fig. 3a) is a 761-residue-long, a helix-rich structure, the chain A of bacteriophage phi6 capsid (EMD-2364; PDB ID: 4btg). The GAN-modified map had an improved RSCC for all (100%) of the 761 residues in the protein when compared with the experimental map. The average RSCC improved from 0.570 to 0.775 (36.0%). The increase of RSCC was achieved for other structure classes, too, as exemplified in Fig. 3b for a β-class protein and in Fig. 3c for an α/β class protein. Figure 3b is for the grapevine fanleaf virus complex (504-residue long, EMD-3246, PDB ID: 5fojB), 99.4% of residues have increased RSCC, with an average RSCC increase from 0.687 to 0.781. For the chain D of membrane-embedded motor of a eukaryotic V-ATPase (EMD-8409, PDB ID: 5tj5) shown in Fig. 3c, an RSCC increase was observed for 97.7% of the residues. The last example (Fig. 3d) is the map that showed the largest decrease of average RSCC. It is a 6 Å map of trimeric HIV-1 envelope glycoprotein (EMD-2484, PDB ID: 4cc8L), where RSCC dropped for 90.4% of the residues which made the average RSCC decreased from 0.539 to 0.330. This is perhaps due to the low map resolution of 6.0 Å, the lowest end of the maps in this study. The training set of 86 maps only included 4 maps at a resolution of 5.5 Å or worse. Corresponding TM-score of MAINMAST models only slightly dropped from 0.648 to 0.643 whereas coverage (Cα atoms within 3 Å) slightly increased from 0.613 to 0.621 (Phenix did not run because the protein sequence included unknown amino acids).

### 3.2 Examples of changes in density maps

**Figure 4.**
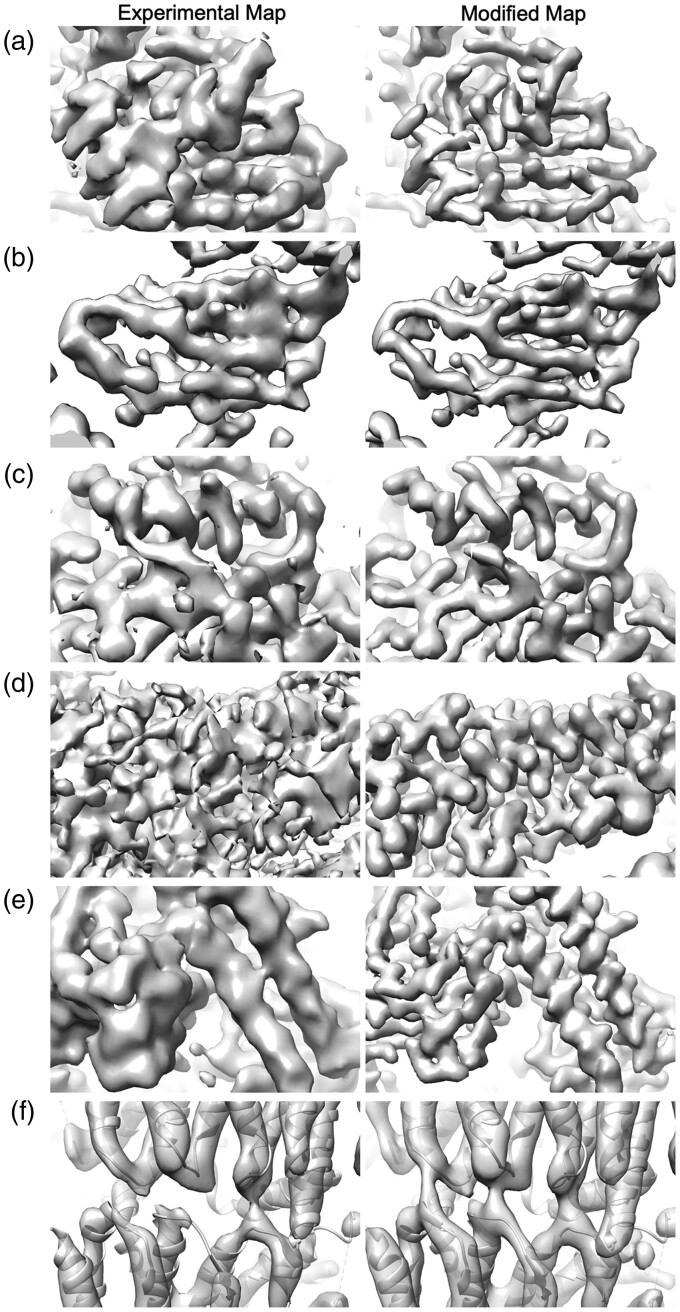
Examples of EM maps before and after applying EM-GAN. In each example, the left figure is the experimental EM map; and the right figure shows the modified map by EM-GAN. The experimental map and the GAN-modified maps are visualized at an equivalent contour level post density normalization to each other. A larger area of the maps is provided in Supplementary Fig. S1. Panels (a–e) are examples with clear visible improvement in the modified maps while panel (f) is the opposite case. (a) EMD-5623 (resolution: 3.3 Å). PDB: 3J9I. The correlation of the experimental map to the simulated map was 0.652, which was improved to 0.726. (b) EMD-8644 (res.: 4.4 Å), PDB: 5V8M. The correlation of the exp. map and the modified map to the simulated map were 0.882 and 0.892. (c) EMD-8624 (res.: 3.4 Å), PDB: 5UZ9. The cc. of the exp. map and the mod. map to the simulated map were 0.64 and 0.793, respectively. (d) EMD-6479 (res.: 3.5 Å), PDB: 3JCK. The cc. of the exp. map, 0.654; the mod. map: 0.689. (e) EMD-3378 (res.: 4.35 Å), PDB: 5FYW. The cc. of the exp. map, 0.838; the mod. map: 0.903. (f) EMD-3672 (res.: 5.7 Å), PDB: 5NP1. The cc. of the exp. map, 0.916; the mod. map: 0.886.

Next, we see how the change by EM-GAN visually impacted density maps in examples (Fig. 4). For each example, two panels are shown: the starting experimental EM map (left), and the modified EM map by EM-GAN (right). For all cases, the density values in both maps were normalized to each other. Figures of larger regions from the same maps with are provided in Supplementary Fig. S1. Figure 4a–e are examples that the modified maps increased the map cross-correlation to the simulated maps and have visible improvements. The first two panels, 4a and 4b are examples of β-sheets. In both cases, strands in β-sheets are more clearly separated in the modified maps, which would help structure modeling. Figure 4c, d are examples of α helices, where the modified map shows more distinct α-helix pitches and sidechains. Figure 4e shows an example where helix densities that were incorrectly connected have been rectified. Figure 4f is an opposite case, where the modified map lowered the cross-correlation to the corresponding simulated map (Fig. 2b). In the modified map, the density of separate loops is merged in several places.

### 3.3 Quality of structure models

We will now discuss how the modified maps enhance the quality of building structural models in practice. For this purpose, we utilized two protein structure modeling software programs: Phenix and MAINMAST. The modeling by Phenix and MAINMAST was performed for a density region in each map that was segmented out for single protein chain structures using the UCSF Chimera’s “zone” tool because these two methods need manual intervention for modeling multi-chains. The modeling was performed for all 65 maps in the testing set.

**Figure 5.**
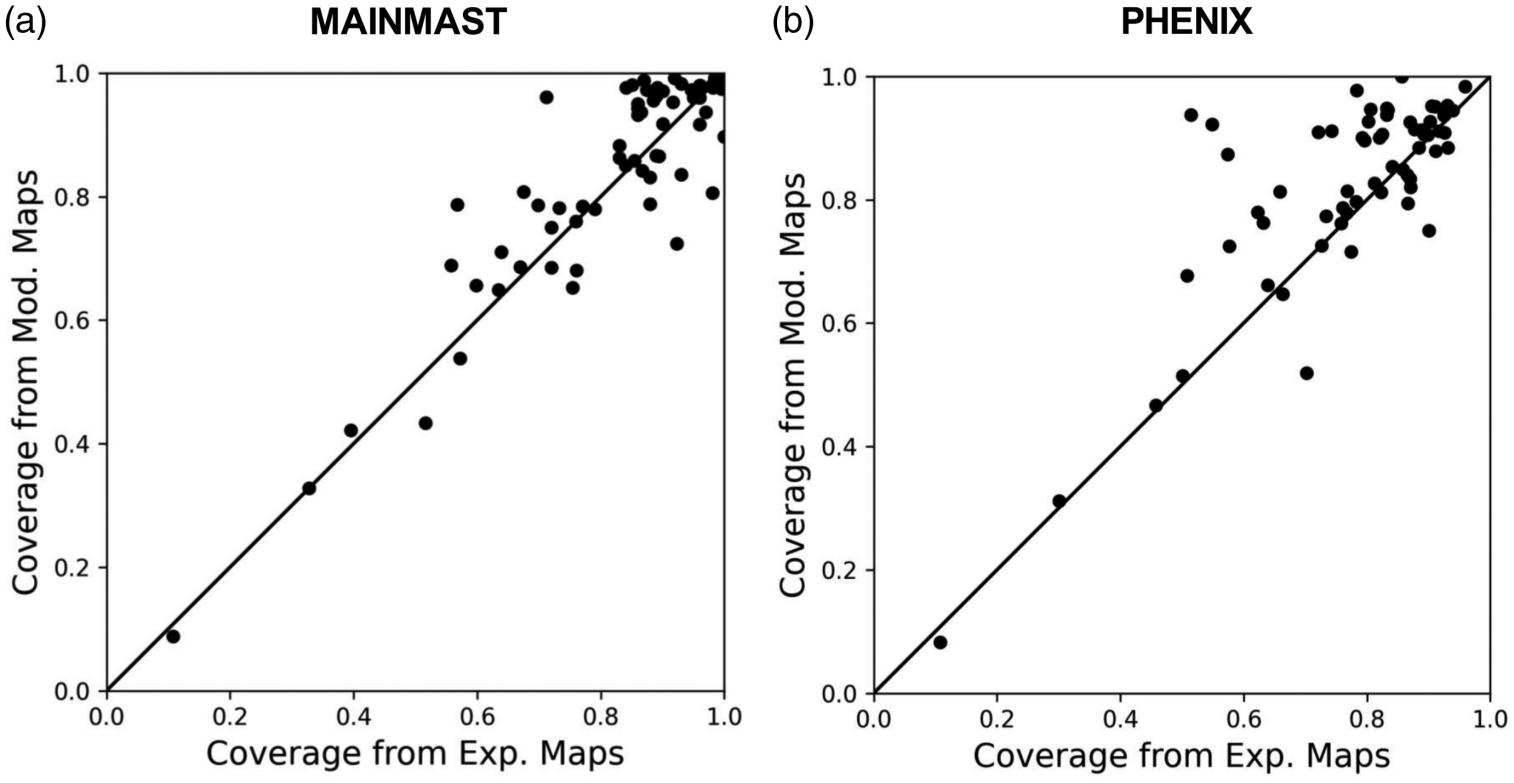
(a, b) Change of the model coverage by two de novo modeling methods, MAINMAST and Phenix. Coverage within 3.0 Å is compared for models generated from experimental maps and GAN-modified maps. (a) Models by MAINMAST. The numbers of cases that improved/tied/worsened by using GAN-modified maps were 42/4/19, respectively. (b) Models by Phenix. The numbers of cases that improved/tied/worsened by using GAN-modified maps were 46/2/15, respectively. There were two maps, EDM-2484 and EMD-8581, for which Phenix did not run because the protein sequence of the maps includes unknown amino acids (which are denoted as X in the PDB files).

The coverages of structure models were calculated using the chain_comparison tool in Phenix (Fig. 5). The coverage is defined as the fraction of Cα atoms in a protein modeled within 3 Å to the native as used in previous works (Baker *et al.* 2012, Terashi and Kihara 2018, Terwilliger *et al.* 2018). For both methods, coverage improved for most of the cases by using modified maps. An improvement or majority of equal coverage was observed for 46 (70.8%) and 48 (73.8%) cases by MAINAST and Phenix, respectively. The largest coverage increase was observed for EMD-8771, which improved from 0.712 to 0.961 (32.7%) in MAINMAST. For Phenix models, the largest increase was by an even larger margin, from 0.549 to 0.922 (67.9%) for EMD-8001. In Supplementary Fig. S2, we further computed RMSD of the structure models. When modified maps by EM-GAN were used, more residues were placed in the protein models by both modeling methods within cutoff values of 3, 5, and 8 Å from amino acids in the native structure. Moreover, despite the inclusion of more residues in the models, the average RMSD of the models were smaller (Supplementary Fig. S2). To evaluate the similarity of models in entirety, we computed TM-score between generated structure models and the corresponding native structures (Supplementary Fig. S3). Overall, we find that the trend aligns with coverage with an improvement, or equal TM-score observed for 50 (76.9%) and 52 (80%) GAN-modified models generated by MAINMAST and Phenix, respectively. Further, in Supplementary Fig. S4, we examined RSCC of the native structures to the modified maps and compared with RSCC of the structure models generated from the modified maps. Overall, the native structures showed a higher RSCC than the structure models indicating hallucinations that led to incorrect modeling is unlikely.

**Figure 6.**
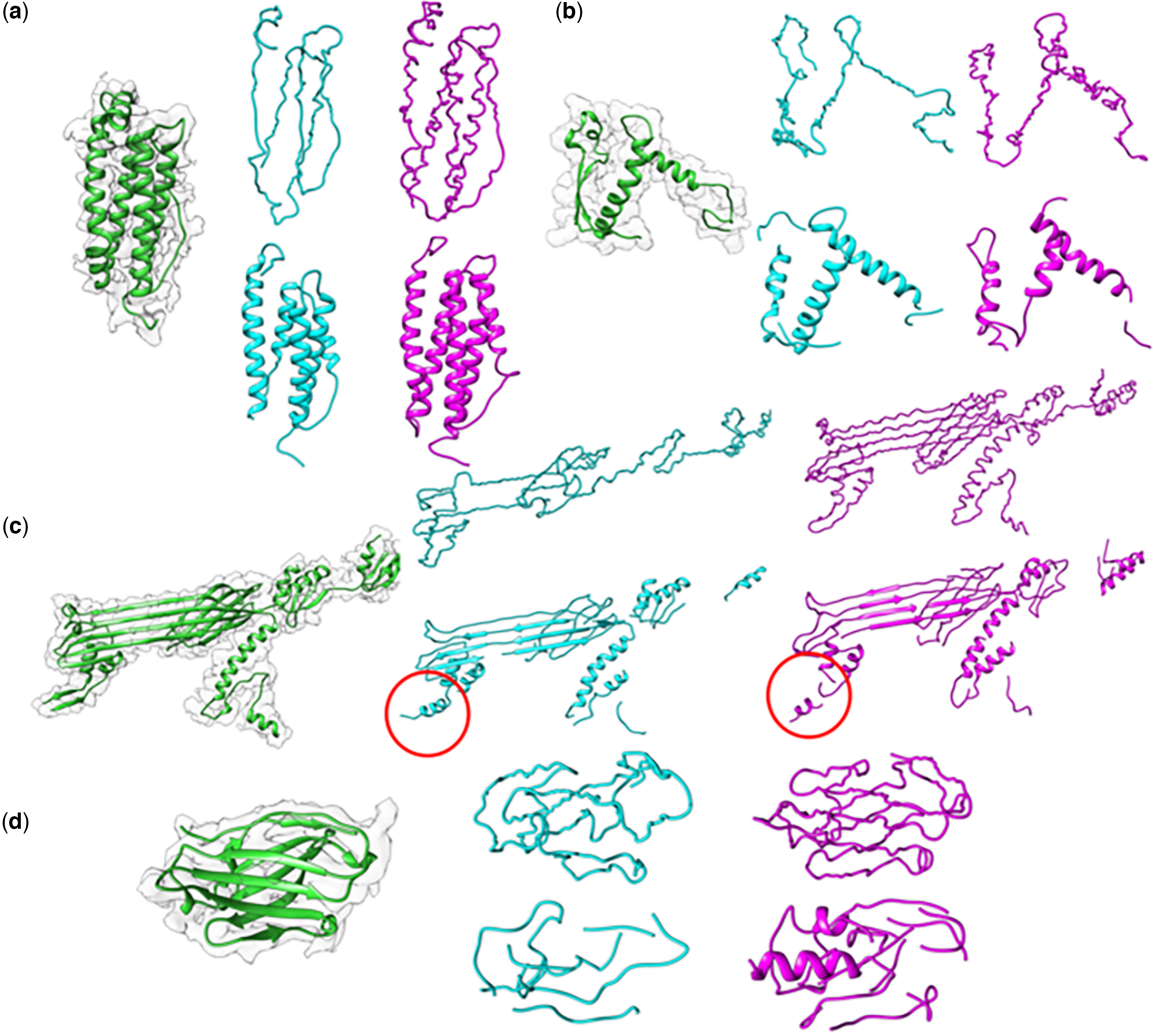
Examples of main-chain models by both MAINMAST and Phenix. These results are from automated runs of main-chain tracing. Thus, in a practical scenario, the models further need sequence mapping, full-atom building, and refinement. Each panel has five subpanels: Leftmost subpanel, the experimental map superimposed with the corresponding protein structure in green. On the right, the upper row shows MAINMAST models, while the lower row shows Phenix models. Cyan, models from original experimental maps; magenta, models from the GAN-modified maps. Local structures mentioned in the text are highlighted with red circles. (a) The 174-residue-long chain O of horse spleen apoferritin (EMD-2788; PDB ID: 4V1W). The MAINMAST main-chain model/the Phenix model generated from the original map had coverage within 3 Å of 0.547/0.876, respectively. Models from the modified map by MAINMAST/Phenix had coverage of 0.959/0.882, respectively. (b) Ebola surface glycoprotein, GP2 (EMD-8242, PDB ID: 5KEN-M), 113 residue-long. Coverage of MAINMAST/Phenix from the original map: 0.761/0.726. From the modified map: 0.681/0.726. (c) The type II secretion system, chain J (EMD-6677, PDB ID: 5WQ9-J), 493 residue-long. Coverage of the MAINMAST/Phenix from the original map: 0.473/0.633. From the modified map: 0.746/0.692. (d) Heavy chain of NIH antibody 3BNC117 (EMD-8644, PDB ID: 5V8M-H), 111 residue-long. Coverage of the MAINMAST/Phenix from the original map: 0.865/0.721. From the modified map: 0.937/0.910.

In Fig. 6, we show several examples of models generated from original and GAN-modified maps by MAINMAST and Phenix. For the comparison of the two methods, the structure modeling software was run without any manual intervention. In each panel in Fig. 6, the native structure in the original map (magenta), a model generated from the original map (green), and a model from the GAN-modified map (cyan) are shown from left to right. The upper row displays the MAINMAST models, while the corresponding Phenix models are shown in the lower row.

The first example (Fig. 6a) is modeling for a 174-residue-long α helical protein. The coverage of the MAINMAST models increased by 0.412, from 0.547 to 0.959, when using the modified map, compared to the models generated from the original map. The use of the modified map allowed MAINMAST to correctly trace all helices, while the model from the original map missed one helix. Additionally, the modified map led to substantial improvement in the modeling of helical pitches due to the enhancement of local density in the map. The Phenix models in the bottom row have helices with correct pitches. The Phenix model generated from the original map has a break in the chain and made a helix shorter than the native, but they were fixed when the modified map was used. The next example in Fig. 6b shows a case where the modeling did not benefit from the use of the modified map. This protein has a two-stranded β-sheet on the left connected with two helices on the right. For this map, EM-GAN made the map worse. This was reflected in the modeling results by both MAINMAST and Phenix as they saw essentially no improvement. Neither method could trace the β-sheet correctly, instead placing an α-helix in the middle of the two strands (Phenix) or intersecting two strands (MAINMAST). MAINMAST dropped the coverage from 0.761 to 0.681 by missing a longer loop region on the left top of the figure.

In Fig. 6c, a substantial improvement in the MAINMAST models was also observed in the modeling of a subunit from the Type II secretion system. The MAINMAST model generated from the modified map has the correct topology for the large β-sheet domain and a long helix in the middle of the structure. Phenix also generated improved models when given the modified map, connecting fragments and exhibiting increased coverage. Phenix made a small, incorrect helix at the left bottom of the structure (red circle), which remained in the GAN-modified-map model. Figure 6d shows models for an antibody heavy chain. In this case, the MAINMAST results substantially improved when using the modified map, where the mainchain was almost perfectly traced except for a missing connection on the right side of the structure. On the other hand, the two Phenix models were fragmented, and moreover, the modified model contained two helices that were misassigned.

### 3.4 Comparison with related methods

Lastly, we compared EM-GAN with OPUS-SSRI (Luo *et al.* 2021) and DeepEMhancer (Sanchez-Garcia *et al.* 2021), existing EM map sharpening methods. The comparison results with OPUS-SSRI are detailed in Supplementary Table S1. EM-GAN outperformed OPUS-SSRI in 6 out of the 7 maps using the d_fsc_model (FSC = 0.143) metric. The largest resolution improvement seen for a map was with EMD-2824, which improved by 0.77 Å (from 3.91 to 3.14 Å). In comparison with DeepEMhancer, EM-GAN showed better resolution estimates than DeepEMhancer for 14 out of 17 cases using d_model metric and 10 out 16 cases using d_fsc_model (FSC = 0.143) metric (Supplementary Table S2). Improvements in protein structure modeling were also examined for maps processed by DeepEMhancer and EM-GAN. We see that EM-GAN outperformed DeepEMhancer for 12 out of the 17 cases with MAINMAST models and 13 out of the 17 cases with Phenix models (Supplementary Fig. S5). In Supplementary Fig. S6, we show examples that compare modified density maps generated by EM-GAN and DeepEMhancer. As shown in panels a, b, c, we observed that maps by EM-GAN have higher density coverage for loop, helix pitches, and protein side-chains. On the other hand, given that DeepEMhancer was trained to sharpen EM maps, we observe sharper surface densities in its modified map in panel d.

## 4 Discussion

In this work, we developed EM-GAN, a 3D DL-based framework which produces modified cryo-EM maps that lead to better downstream protein structure modeling performance. This is the first work successfully applying GAN for improved structure modeling from 3D cryo-EM maps.

At this juncture, it would be appropriate to mention the limitations of the current work. Since the GAN was trained on simulated EM maps generated from protein structures, its modification of maps may not effectively handle densities that come from other molecules such as ligands or water molecules in the map, if present. In the future, with an increase in the number of experimental maps available, modification of maps that takes into account other molecule types will also be feasible. The current framework can also be extended to maps at higher or lower resolutions by training the network with an appropriate dataset. One potential concern when using generative models is hallucinations, where generated data is artificially modified that can lead to misinterpretation of input data. In light of this concern, we would like to emphasize that the purpose of EM-GAN is solely to assist in protein structure modeling, which is the common end product of an EM map. The modified maps generated by EM-GAN are intended to be used only as intermediates for this purpose and should not be interpreted in other ways. It is strongly recommended that experimentalists carefully compare and validate the structure models generated from the modified map with the original experimental map and consider refining the models using the original map if necessary.

## Supplementary Material

btad494_Supplementary_Data

## Data Availability

EM-GAN is freely available in open source form at https://github.com/kiharalab/EM-GAN and at Google Colab https://tinyurl.com/3ccxpttx. The dataset used are from public databases, PDB and EMDB.
