# Supplementary material for: Enhancing cryo-EM maps with 3D deep generative networks for assisting protein structure modeling
Source: Bioinformatics. 2023 Aug 7;39(8):btad494. doi: 10.1093/bioinformatics/btad494 (PMC10444963; doi:10.1093/bioinformatics/btad494)
Supplement: btad494_Supplementary_Data [file btad494_supplementary_data.pdf]

Supplementary Information for

Enhancing Cryo-EM Maps With 3D Deep Generative Networks For Assisting Protein Structure Modeling

Sai Raghavendra Maddhuri Venkata Subramaniya[1], Genki Terashi[2], and Daisuke Kihara*[2,1]

[1] Department of Computer Science, Purdue University, West Lafayette, IN, 47907, USA

[2] Department of Biological Sciences, Purdue University, West Lafayette, IN, 47907, USA

* Contact: dkihara@purdue.edu

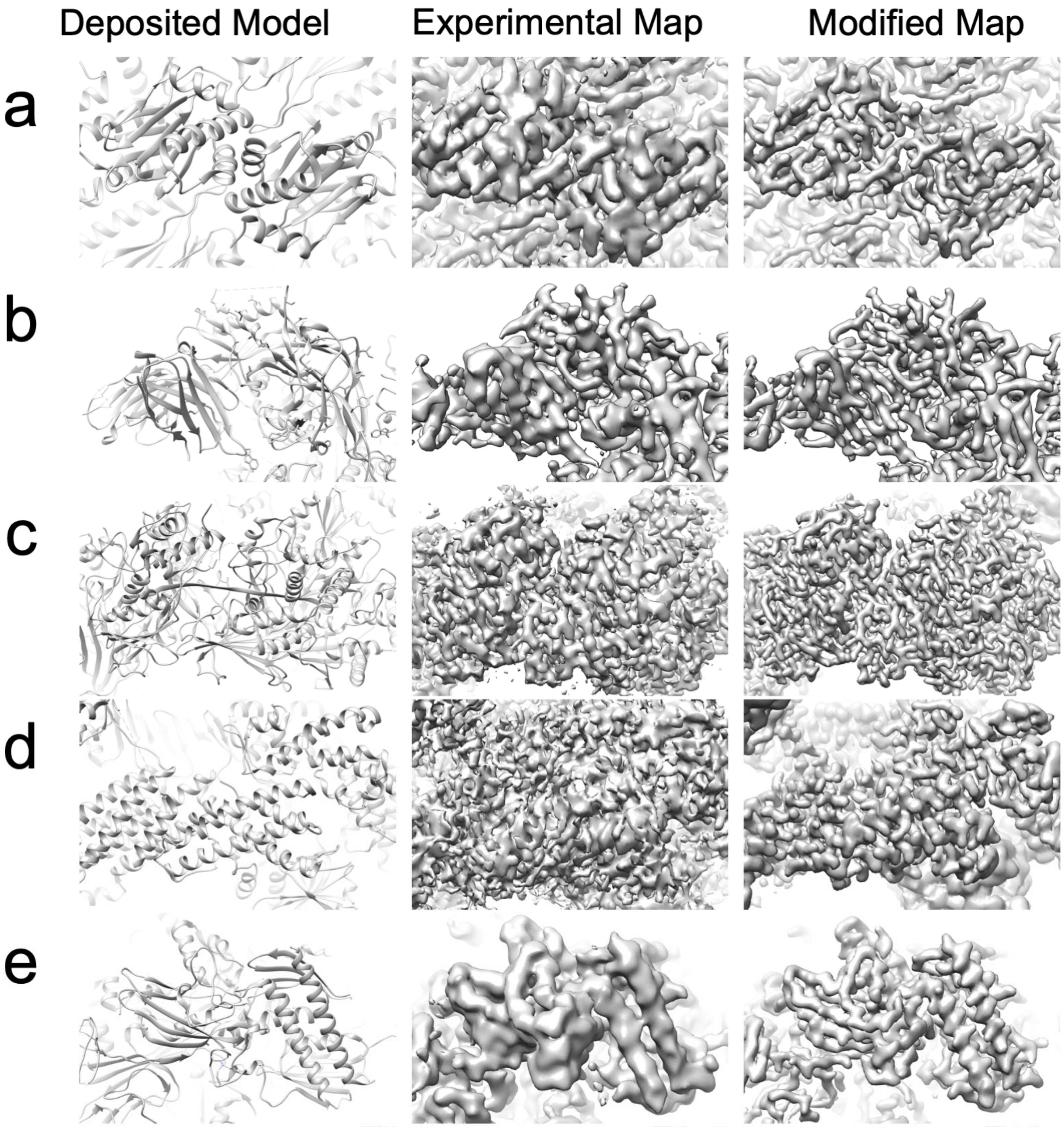
Deposited Model
Experimental Map
Modified Map
a
b
c
d
e

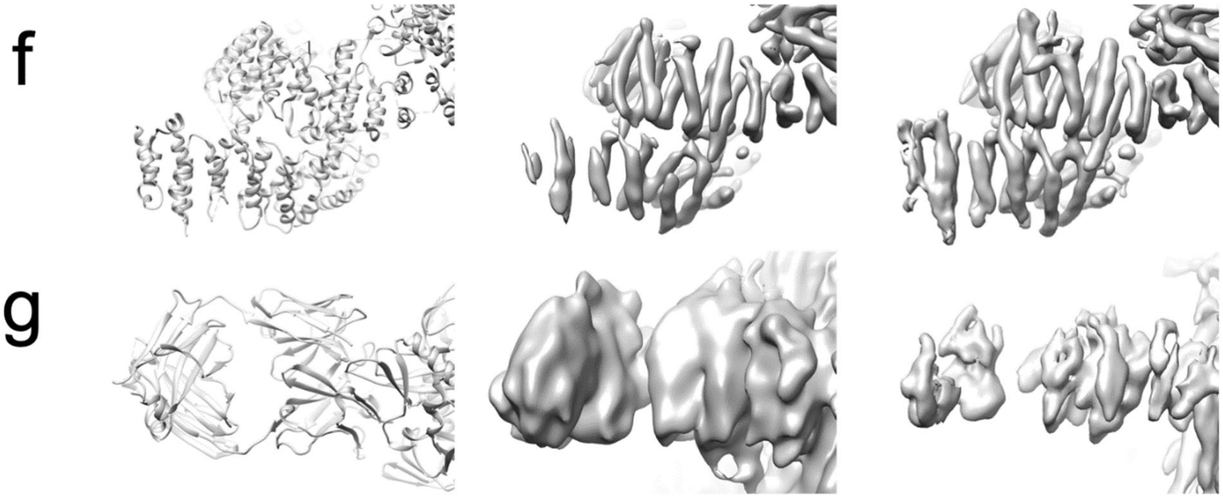

Supplementary Figure S1. Examples of EM maps before and after applying EM-GAN.

For each example, three panels are shown. Left, the deposited atomic model. Middle, the experimental map. Right, the modified map by EM-GAN. The experimental map and the GAN-modified maps are visualized at an equivalent contour level post density normalization to each other.

Panels *a* to *e* are cases that showed improvement by applying EM-GAN. These maps are the same maps as shown in panels *a* to *e* in Figure 4 in the main text. In Figure 4, we further magnified the maps. The density of protein main-chains and sidechains are more distinct in modified maps.

a, EMD-5623 (resolution: 3.3 Å). PDB: 3J9I. The contour level in the experimental map is 0.15. the correlation between the exp.map/the simulated map: 0.652; the modified map/sim. map: 0.726.
b, EMD-8644 (resolution: 4.4 Å), PDB: 5V8M. The contour level in the exp. map is 0.045. Exp./Sim.: 0.882, Mod./Sim.: 0.898.
c, EMD-8624 (resolution: 3.4 Å), PDB: 5UZ9. The contour level in the exp. map is 0.026. Exp./Sim.: 0.640, Mod./Sim.: 0.793.
d, EMD-6479 (resolution: 3.5 Å), PDB: 3JCK. The contour level in the exp. map is 0.02. Exp./Sim.: 0.654, Mod./Sim.: 0.689.
e, EMD-3378 (resolution: 4.35 Å), PDB: 5FYW. The contour level in the exp. map is 0.0285. Exp./Sim.: 0.838, Mod./Sim.: 0.903.

Panels *f* and *g* are opposite examples where modified maps deteriorated correlation to the simulated map. Panel *f* is the same example as shown in Figure 4. Panel g is new example in the Supplement and not included in Figure 4.

f, EMD-3672 (resolution: 5.7 Å), PDB: 5NP1. The contour level in the experimental map is 0.066. Exp./Sim.: 0.916, Mod./Sim.: 0.886. In the modified map, densities of neighboring chain positions are connected. See Figure 4e for a magnified map.
g, EMD-2484 (resolution: 6.0 Å), PDB: 4CC8. The contour level in the experimental map is 0.03. Exp./Sim.: 0.813, Mod./Sim.: 0.630. Although the modified map has more distinctive in individual parts, it is not particularly more informative for protein modeling. It also has some disconnections.

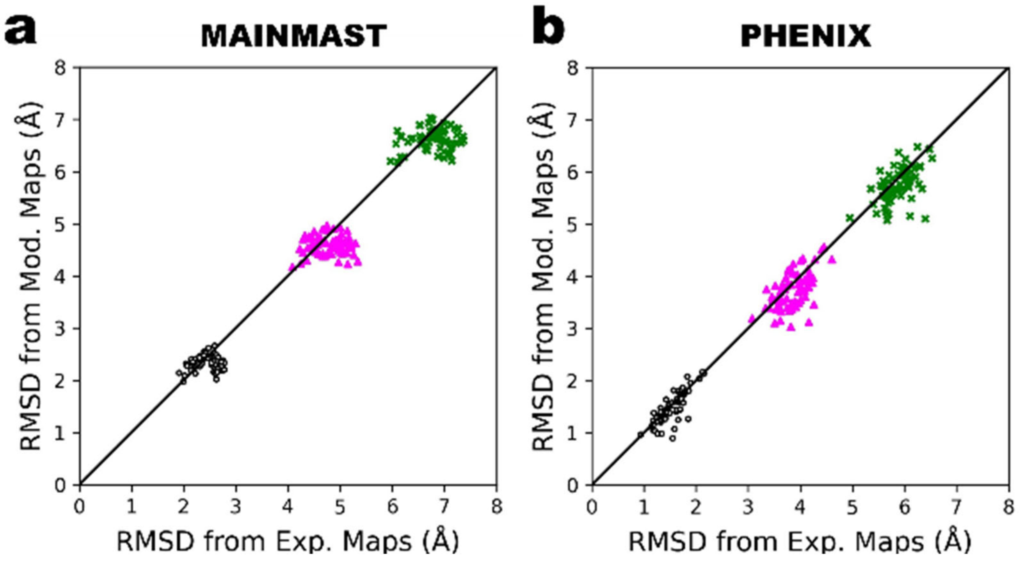

Supplementary Figure S2. RMSD of models built from experimental maps and GAN-modified maps.

a, MAINMAST; **b**, PHENIX was used for modeling. Black, RMSD computed for residues in a model that were within 3 Å to the correct position. Magenta, RMSD using residues within 5 Å; green, RMSD using residues within 8 Å. Phenix chain_comparison tool was used for the computation.

When MAINMAST was used for modeling, structure models of 41, 49, 49 over 65 maps increased or had the same number of correctly placed residues within 3, 5, 8 Å, respectively, over structure models derived from the original experimental maps. The average number of residues included in MAINMAST models were 128, 141, 162, for 3, 5, 8 Å cutoffs, respectively, when modified maps were used, which were 6.67%, 13.7%, and 8.0% increase when compared with models from the experimental maps.

In terms of RMSD, 34, 44, and 43 models improved by using modified maps, when residues within 3, 5, 8 Å cutoff were used. The average RMSD of models including residues within 3, 5, 8 Å cutoff were 2.34 Å/2.44 Å, 4.55 Å/4.78 Å, 6.52 Å/6.78 Å¸ when modified/experimental maps were used.

With Phenix, structure models of 46, 47, 51 over 65 maps increased or had the same number of correctly placed residues within 3, 5, 8 Å, respectively, over structure models derived from the original experimental maps. The average number of residues included in Phenix models were 131, 151, 167, for 3, 5, 8 Å cutoffs, respectively, when modified maps were used, which were 9.17%, 16.2%, and 16.0% increase when compared with models from the experimental maps.

In terms of RMSD, 37, 41, 46 models improved by using modified maps, when residues within 3, 5, 8 Å cutoff were used. The average RMSD of models including residues within 3, 5, 8 Å cutoff were 1.49 Å/1.54 Å, 3.74 Å/3.88 Å, 5.74 Å/5.87 Å¸ when modified/experimental maps were used.

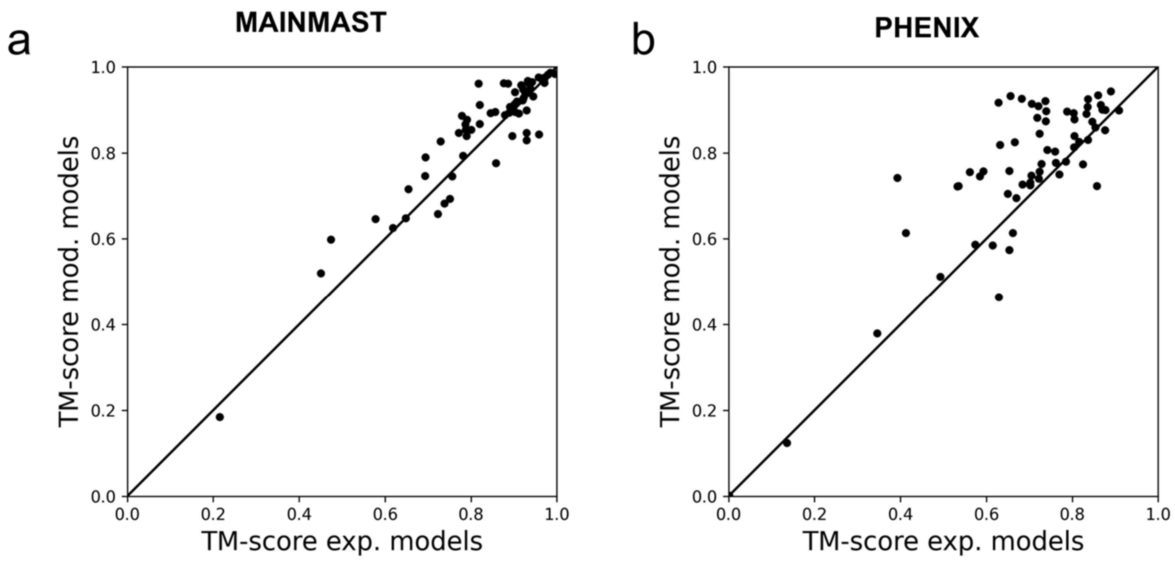

Supplementary Figure S3. TM-score of models built from experimental maps and GAN-modified maps.

TM-score of models from MAINMAST and Phenix generated from experimental and GAN-modified maps **a,** models by MAINMAST. The number of cases where modified models were better/worse than experimental models are 50/15, respectively. The average TM-score of models from modified maps is 0.858, average TM-score of models from experimental maps is 0.839. **b,** models by Phenix. The number of cases where modified models were better/worse than experimental models are 52/13, respectively. The average TM-score of models from modified maps is 0.767, average TM-score of models from experimental maps is 0.696.

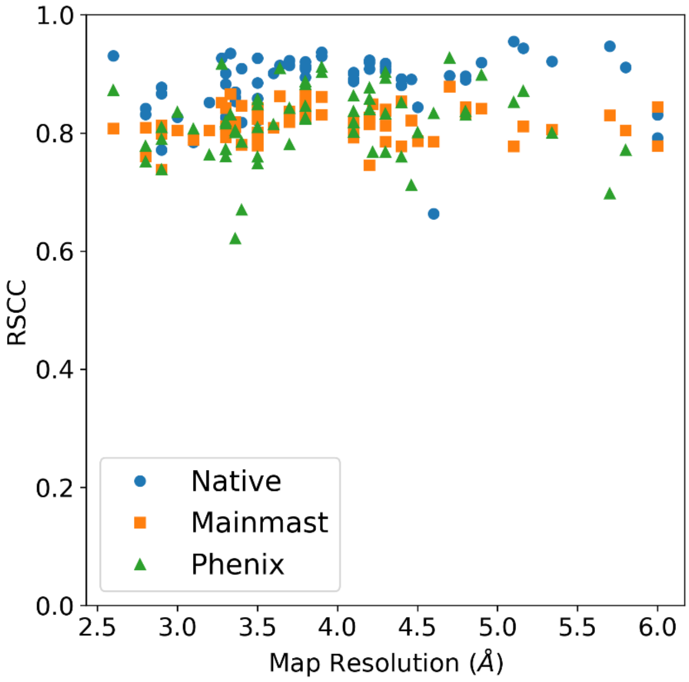

Supplementary Figure S4. RSCC of structure models and the native structure relative to the modified map relative to the map resolution. Blue circles, native structures; orange squares, structure models by Mainmast built from the modified maps; green triangles, structure models by Phenix from the modified maps. RSCC was computed for the modified map region.

Overall, the native structures have the highest correlation to the modified maps than the structure models generated by Mainmast and Phenix, except for one map (EMD-6489, resolution: 4.6 Å).

For the map of EMD-6489, EM-GAN did not work well and decreased the density of locally low-resolution regions of the map and thus the overlap to the native structure is substantially reduced. The correlation to the native structure was lowered to the level below the correlation of the Mainmast and Phenix models built from the modified map. Structure models generated from the modified maps always have a correlation around 0.8 for any cases, as shown in this plot.

For this map, structure modeling was not successful from the original map: TM-Score of the Mainmast and Phenix models built from the original (experimental) map were 0.22 and 0.14, respectively (the outlier at the left bottom corner of Supplementary Fig. S3). With the modified map, modeling did not improve: TM-Score of models from the modified map were 0.19 (Mainmast) and 0.12 (Phenix).

Thus, overall modified maps by EM-GAN have a high correlation to the native structures and would not induce "hallucination" that could lead to wrong structure models.

Supplementary Table S1. Comparison of resolution estimates between modified EM maps generated by EM-GAN and OPUS-SSRI.

EMPAIR / EMDB ID	PDB	OPUS-SSRI	EM-GAN
EMPAIR-100017 / EMD-2824	3I3E	3.91	**3.14**
EMPAIR-10002 / EMD-2275	3U5B	**3.89**	3.92
EMPAIR-10097 / EMD-8731	3WHE	3.72	**3.69**
EMPAIR-10126 / EMD-7132	6BQR	2.93	**2.66**
EMPAIR-10099 / EMD-8639	5V7V	4.25	**4.09**
EMPAIR-10254 / EMD-0594	6O1P	2.76	**2.66**
EMPAIR-10123 / EMD-7095	6BGI	3.14	**2.94**

Resolution estimates (Å) computed with the phenix.mtriage tool (http://www.phenix-online.org/documentation/ reference/mtriage.html) for maps generated by EM-GAN and by OPUS-SSRI are shown. The smaller value was highlighted in bold. The dataset and resolution estimate values of OPUS-SSRI were taken from the test dataset used in the OPUS-SSRI paper detailed in their Supplementary Table S1. We also tried installing OPUS-SSRI software using the instructions at https://github.com/alncat/cryoem but were not successful because of build errors.

We ran EM-GAN, for these 7 maps and computed map vs model correlation using the d_fsc_model (FSC=0.143) metric from phenix.mtriage tool.

Supplementary Table S2. Comparison of resolution estimates between modified EM maps generated by EM-GAN and DeepEMhancer.

EMDB-ID	Original	d_model		d_fsc_model	
		EM-GAN	DeepEMhancer	EM-GAN	DeepEMhancer
3663*	5.16/5.90	5.80	**5.50**	**5.08**	5.50
4078*	5.10/5.10	4.90	**4.20**	**3.79**	4.35
5623*	3.30/3.30	**1.50**	2.90	2.02	**1.97**
6479*	3.50/3.50	**1.60**	3.30	**2.19**	2.27
6714*	3.00/3.10	**1.60**	2.90	**2.54**	2.58
8624*	3.40/3.50	**2.10**	3.00	**2.55**	2.96
10649	4.30/4.30	**2.60**	4.00	3.98	**3.94**
10094	3.34/3.40	**1.40**	2.10	1.98	**1.89**
0975	3.10/3.50	**1.80**	3.00	2.70	2.70
0882	3.30/3.40	**1.40**	3.00	**1.88**	2.50
0920	3.40/3.70	**1.80**	3.10	**2.12**	2.77
0510	3.63/3.50	**3.10**	3.40	**2.78**	3.32
0981	2.84/3.20	**1.40**	2.00	1.77	**1.75**
10088	4.20/4.20	**2.30**	3.90	**2.97**	3.55
10350	3.70/4.00	3.70	**3.10**	2.73	**2.67**
10704	4.08/4.10	**3.30**	3.90	**2.67**	2.68
10836	3.30/3.40	**1.80**	2.10	2.08	**2.06**
Avg. Gain	-	1.35	0.57	1.13	0.92

Resolution estimates (Å) computed with the phenix.mtriage tool (http://www.phenix-online.org/documentation/reference/mtriage.html) for maps generated by EM-GAN and by DeepEMhancer are shown. Original shows the map resolution of the experimental maps. Two values are the one stated in EMDB/measured by phenix.mtriage. Results are shown using two output metrics from mtriage : d_model and d_fsc_model (FSC=0.143). Comparing the resolutions by EM-GAN and DeepEMhancer, the smaller value was highlighted in bold.

The six EMDB-IDs marked with * are included in our benchmark set. The rest of the targets used here were selected from recent deposits (after May 2020) to EMDB. All these targets have half-map data available at EMDB. Input to DeepEMhancer for a target map was half-map because it was trained on half-map and that is stated as the preferred input, while the input for EM-GAN is the original deposited map to EMDB, as was used for the rest of the current study.

Avg. gain shows the average improvement of the resolution relative to the mtriage-measured resolution of the original map.

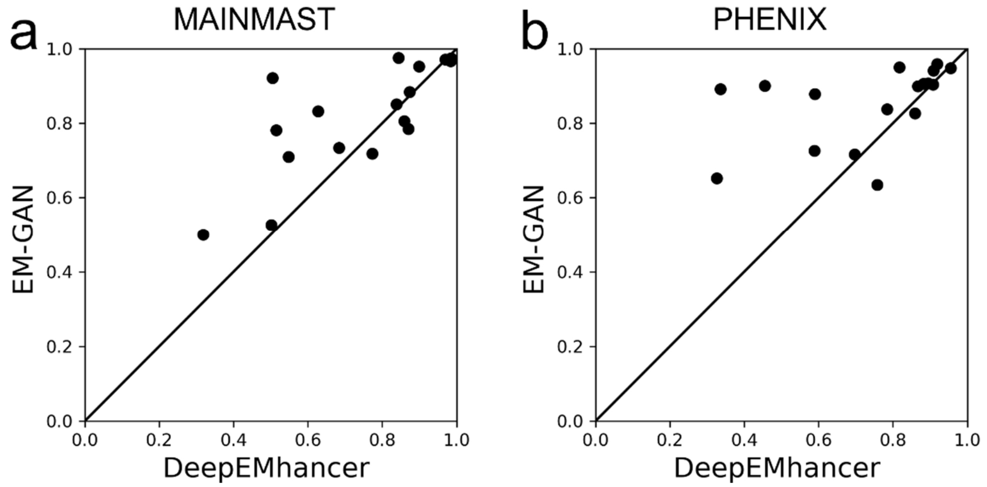

Supplementary Figure S5. Comparison of modeling results by MAINMAST and Phenix using modified EM maps generated by EM-GAN and DeepEMhancer.

Model coverage by two de novo modelling methods, MAINMAST and Phenix. Coverage within 3.0 Å are compared for models generated from maps modified by DeepEMhancer and EM-GAN. **a,** models by MAINMAST. The number of cases where EM-GAN was better/worse than DeepEMhancer are 12/5, respectively. The average coverage of models from EM-GAN modified maps is 0.817, average coverage from DeepEMhancer modified maps is 0.741 (an improvement of 10.3%, P-value: 0.035). **b,** models by Phenix. The number of cases where EM-GAN was better/worse than DeepEMhancer are 13/4, respectively. The average coverage of models from EM-GAN modified maps is 0.852, average coverage from DeepEMhancer modified maps is 0.738 (an improvement of 15.4%, P-value: 0.022).

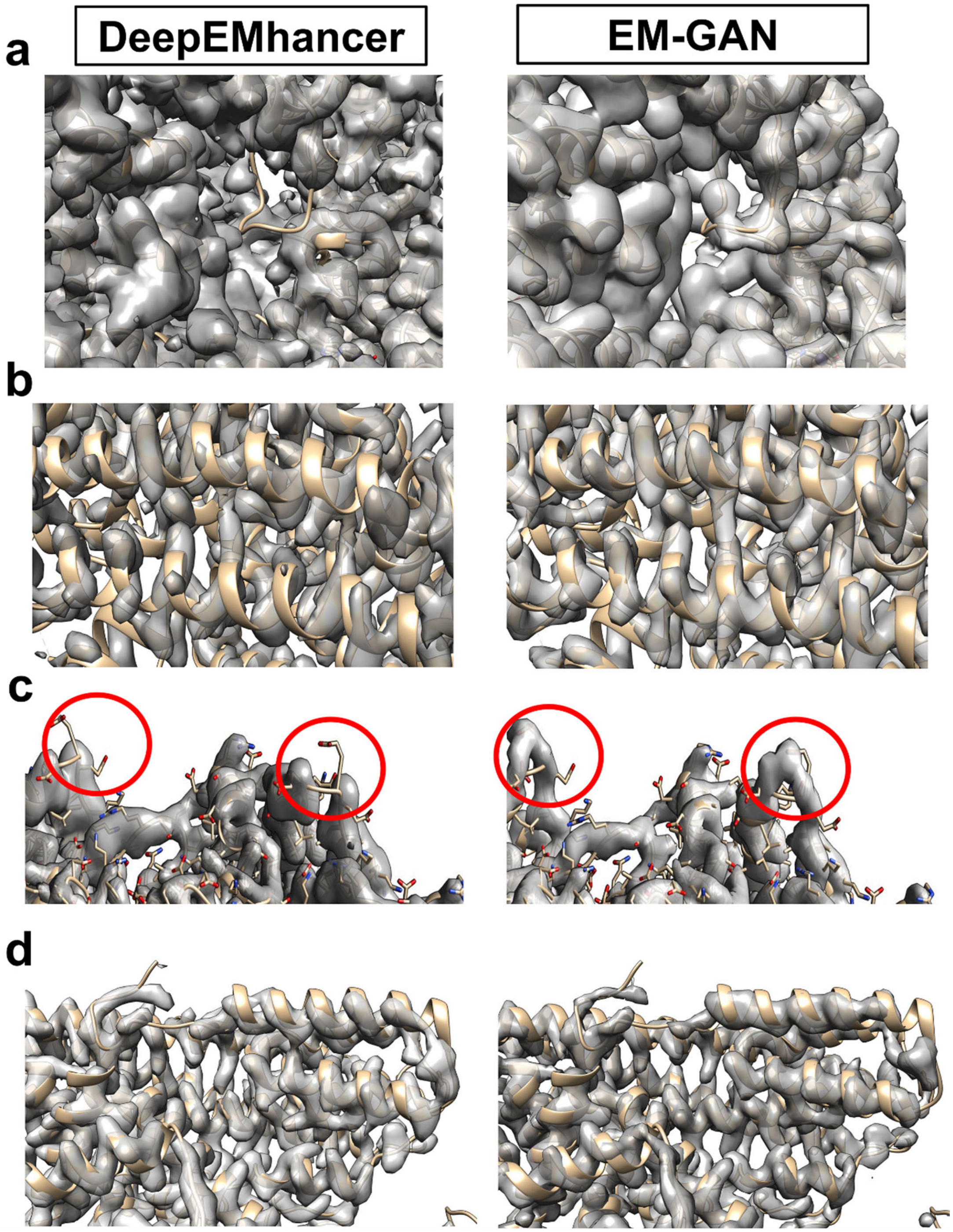

Supplementary Figure S6. Comparison of modified maps generated by EM-GAN and DeepEMhancer

Comparison of maps modified by DeepEMhancer (left) and EM-GAN (right). **a, b, c** are examples where EM-GAN showed smaller (better) d_model and d_fsc_model values than DeepEMhancer. **d** is the opposite case where the map modified by DeepEMhancer showed lower d_model and d_fsc_model values than EM-GAN.

a, EMID: 0510. d_model metric in Å for EM-GAN modified map: 3.10, DeepEMhancer modified map: 3.40. d_fsc_model metric in Å for EM-GAN modified map: 2.78, DeepEMhancer modified map: 3.32. The figure shows a missing loop in the DeepEMhancer-refined map, which is covered by the density in the map by EM-GAN.

b, EMID: 10704. d_model, EM-GAN modified map: 3.30, DeepEMhancer modified map: 3.90. d_fsc_model, EM-GAN modified map: 2.67, DeepEMhancer modified map: 2.68. The EM-GAN modified map have better density coverage for the two helices.

c, EMID: 8624. d_model, EM-GAN modified map: 2.10, DeepEMhancer modified map: 3.00. d_fsc_model, EM-GAN modified map: 2.55, DeepEMhancer modified map: 2.96. A higher density coverage for side-chains at the surface are observed in the EM-GAN modified map.

d, EMID: 10350. d_model, EM-GAN modified map: 3.70, DeepEMhancer modified map: 3.10. d_fsc_model, EM-GAN modified map: 2.73, DeepEMhancer modified map: 2.67. The map modified by DeepEMhancer showed sharper density at helices.

Supplementary Table S3. Computational Cost for EM-GAN inference.

Map	Voxel size (in Å^3)	Inference Time
8728	131*131*131	5 mins 11 secs
4078	260*260*260	16 mins 18 secs
8148	348*348*348	29 mins 36 secs

Summary of computational inference times for EM-GAN shown for maps of different sizes.
